# Patterns in Curated Celebrity Mortality Data: An Exploratory Analysis of Cardiovascular Disease Representation and Dataset Bias

**DOI:** 10.7759/cureus.108973

**Published:** 2026-05-16

**Authors:** Mohamed Ahmed Atwa, Mohammed Ismail Eldesouki Mohammed Maarouf, Bassant Ahmed Atwa, Amira Elsayed Al Anwar

**Affiliations:** 1 Medical School, Kasr Al Ainy Faculty of Medicine, Cairo University, Cairo, EGY; 2 Cardiology and Vascular Medicine Department, Mansoura Specialized Hospital, Mansoura, EGY

**Keywords:** cardiovascular disease, celebrity mortality, dataset bias, descriptive epidemiology, media epidemiology, reporting bias

## Abstract

Background: Publicly reported celebrity deaths receive substantial media attention and may influence how individuals perceive disease burden. However, datasets constructed from publicly available sources are often curated and may not reflect underlying epidemiological distributions. Understanding how dataset construction influences observed mortality patterns is therefore important.

Objective: To present a purposively curated and openly documented dataset of publicly reported celebrity deaths as a worked methodological illustration of how dataset construction, selection, and reporting practices may shape apparent mortality distributions in media-visible data.

Materials and methods: A purposively curated dataset of 164 celebrity deaths was assembled from publicly available English-language sources, principally Wikipedia entries and their referenced material. Cases were selected to include variation in age, reported sex, and cause of death to enable descriptive comparison. Causes of death were classified as cardiovascular or non-cardiovascular using rules defined a priori, and cardiovascular disease (CVD) cases were further subdivided into six International Classification of Diseases (ICD)-aligned subcategories. To assess classification reproducibility, a blinded second coder independently re-classified a random sample of 30 cases. The dataset, the case-level ICD mapping dictionary, and a reproducible analysis pipeline are openly available.

Results: Cardiovascular causes accounted for 84 of 164 deaths in the curated dataset (51.2%); non-cardiovascular causes accounted for 80 deaths (48.8%). Within the CVD group, the largest subcategory was heart failure and other unspecified CVD (33 cases; 39.3%), followed by ischemic heart disease (22 cases; 26.2%), cerebrovascular disease (nine cases; 10.7%), arrhythmia and sudden cardiac death (seven cases; 8.3%), aortic and pulmonary vascular disease (seven cases; 8.3%), and cardiomyopathy and structural heart disease (six cases; 7.1%). The two coders agreed on the binary CVD/non-CVD classification for all 30 cases in the reliability sample (Cohen’s κ = 1.00) and on the six-category subcategory classification for all 18 cases that both coders classified as CVD (Cohen’s κ = 1.00). Cardiomyopathy and structural cases occurred at a notably younger mean age (35.5 years) than other CVD subcategories. CVD deaths overall occurred at older ages than non-CVD deaths and were predominantly observed among males.

Conclusions: The patterns observed in this curated dataset reflect the combined effects of investigator selection, demographic non-comparability between celebrities and the general population, and reporting practices in publicly available sources. The high proportion of non-specific cause-of-death descriptions illustrates an important component of reporting bias in media-visible mortality data.

## Introduction

The media plays a central role in shaping public understanding of health and disease. Through news coverage and digital platforms, information about illness and mortality is communicated to large audiences and can influence how individuals perceive disease risk and public health priorities. This influence is well described through agenda-setting mechanisms, whereby media coverage can shape which health issues are perceived as most important by the public [1]. In addition, exposure to health-related media content has been shown to affect awareness and behavior at the population level [2]. Celebrity health events, in particular, receive widespread attention and may exert a disproportionate influence on public perceptions of disease [3,4].

Celebrity deaths are highly visible events and are frequently reported across media platforms. However, the patterns observed in publicly available or widely circulated reports of celebrity mortality may not reflect underlying epidemiological distributions. Such patterns may be influenced by selective reporting, public interest, and the visibility of specific causes of death. They may also reflect demographic features that distinguish celebrities from the general population, including older age at peak public visibility, greater healthcare access, and occupational exposures specific to public-facing careers. Psychological and social mechanisms, including identification with public figures and amplification through media coverage, may further contribute to differences between perceived and actual disease burden [3,5].

Cardiovascular disease (CVD) remains the leading cause of mortality worldwide, accounting for approximately one-third of all deaths globally [6,7]. Accurate public understanding of its prevalence is therefore essential for effective prevention strategies and health communication. When mortality data are derived from curated or publicly visible sources rather than systematic population-level datasets, the resulting patterns may reflect characteristics of the dataset itself rather than true epidemiological distributions.

Despite the potential for publicly visible mortality data to influence perception, there has been limited quantitative exploration of how dataset construction affects observed mortality patterns. Few studies have examined how curated datasets of celebrity deaths represent major disease categories, such as CVD, or how these representations compare with population-level reference values.

The present study addresses this gap by presenting a purposively curated and openly documented dataset of publicly reported celebrity deaths as a worked methodological illustration. Rather than estimating the true distribution of celebrity mortality or quantifying the contribution of any single bias mechanism, this study describes patterns observed in the dataset to illustrate how dataset construction, selection, and reporting processes jointly influence the interpretation of mortality data derived from publicly available sources. The manuscript’s intended contributions are threefold: (i) the public release of an openly auditable, rule-classified dataset that allows the abstract concerns of selection-bias methodology to be inspected at the level of individual cases; (ii) the explicit operationalization of reporting specificity at two distinct levels (field-level label vs. subcategory-level underlying-etiology specificity) and the demonstration that these two levels can yield substantially different specificity estimates within the same dataset; and (iii) an empirical illustration that publicly available mortality reporting concentrates non-etiologic terminal-syndrome descriptions to a degree analogous to but distinct from the “garbage code” phenomenon in vital-registration data. The dataset, the case-level International Classification of Diseases (ICD) mapping dictionary, an inter-rater reliability record, and a reproducible analysis pipeline are openly available [8].

## Materials and methods

Operational definition and case identification

For the purposes of this study, a celebrity was operationally defined, as an instrument of dataset construction rather than as a definition of celebrity per se, as an individual with a dedicated English-language Wikipedia biographical entry indicating notability in entertainment (film, television, music, comedy), professional sport, literature, or public broadcasting, and for whom a documented cause of death was reported in the entry or its cited references. This definition selects on cultural prominence within one language environment and is systematically biased toward Anglophone, recent, and Western-media-visible figures; comparative analyses across language editions of Wikipedia have shown that the prominence and ranking of public figures differ substantially by cultural context [9]. The restriction to English-language publicly visible sources is, therefore, itself a documented source of bias that both enables the present methodological illustration and limits any claim to external validity; this is acknowledged further in the Limitations.

Cases were identified through English-language Wikipedia biographical entries and related compilations of deaths with documented causes, including Wikipedia’s annual “Deaths in [year]” pages and category indices for deaths by cause. Wikipedia served as a primary index to identify cases and access associated source material, reflecting the type of publicly visible information commonly encountered in media and online environments. Data extraction was conducted between January and April 2026, with the final database closed on April 25, 2026. A retrospective characterization of the source-list categories consulted, and the structure of the inclusion decisions is provided as a supplementary file with the dataset deposit [8]; a prospective case-by-case inclusion log was not maintained during data extraction, and this is acknowledged in the Limitations.

Cases were purposively selected to include variation in age, reported sex, and cause of death, enabling descriptive comparison across categories. The dataset is therefore an exploratory curated sample rather than a systematically sampled or population-representative cohort. No prespecified target sample size was set; case ascertainment continued until further inspection of source lists was yielding cases either already in the dataset or without sufficiently documented cause-of-death information to meet inclusion criteria, at which point recruitment was closed at 164 individuals. The implications of this design for interpretation are addressed in the Discussion and Limitations.

Variables and classification

For each individual, the following variables were recorded: name; reported sex (coded as male or female on the basis of pronouns and identifiers used in the source biographical entry, since direct ascertainment of sex assigned at birth or self-identified gender was not possible from secondary sources); age at death; ethnicity (when available); reported cause of death; cause category (cardiovascular vs. non-cardiovascular); source reference; year of death; occupation or public-figure type; and an indicator of cause-report specificity. The specificity indicator was operationalized at the field level: a cause was flagged as “non-specific” only if the reported description gave no medically meaningful information (e.g., “natural causes” and “long illness”), and as “specific” otherwise (e.g., a named medical condition such as “myocardial infarction” or “heart failure”). This variable, therefore, captures whether any clinical label was reported, not whether that label identified an underlying etiology; the distinction is examined further in the Discussion.

Cardiovascular causes were defined as conditions corresponding to the International Classification of Diseases, Tenth Revision (ICD-10) Chapter IX (I00-I99), and included myocardial infarction, cardiac arrest, heart failure, stroke, aortic dissection, myocarditis, and related vascular diseases. All other causes were classified as non-cardiovascular.

To address the heterogeneity inherent in the broad CVD category, cardiovascular cases were further subdivided into six ICD-aligned subcategories: (i) ischemic heart disease (I20-I25); (ii) cerebrovascular disease (I60-I69); (iii) aortic and pulmonary vascular disease (I26, I71-I72); (iv) cardiomyopathy and structural or congenital heart disease (I42, Q20-Q24); (v) arrhythmia and sudden cardiac death (I46-I49); and (vi) heart failure and other unspecified CVD (I40, I50-I51 and residual codes). When a reported description referenced multiple events, the case was classified by the terminal precipitating cardiovascular event. Cardiac arrest without a specified underlying etiology was assigned to the arrhythmia and sudden cardiac death subcategory, in keeping with its description as a terminal rhythm rather than a distinct etiology. Primary classification was performed by one investigator (M.A.A.) using these a priori rules: the case-level application of the rules - that is, the verbatim reported cause-of-death description, the assigned subcategory, and the corresponding ICD-10 range for every CVD case - is deposited as a supplementary mapping dictionary [8] to permit inspection of every classification decision.

To assess classification reproducibility, a second coder (B.A.A.) independently re-classified a random sample of 30 cases drawn from the dataset using a fixed random seed (seed = 20260513, reproducible via the analysis pipeline deposited with the dataset [8]) and applying the same a priori rules, blinded to the original classifications. Inter-rater agreement on the binary CVD/non-CVD classification was perfect (30 of 30 cases in agreement; Cohen’s κ = 1.00); inter-rater agreement on the six-category CVD subcategory classification was also perfect, restricted to the 18 cases that both coders classified as CVD (18 of 18 cases in agreement; Cohen’s κ = 1.00). The case-level re-classification record is deposited alongside the dataset [8].

The final dataset comprised 164 individuals and is openly available on Zenodo [8].

Dataset validation

A multi-stage validation process was applied to improve internal consistency and data quality. Structural checks confirmed the presence of all required variables and appropriate data formats. Automated procedures standardized categorical variables and verified plausible ranges for numeric variables such as age. Duplicate detection ensured that each individual was uniquely represented. Source verification was conducted using the referenced material associated with each entry; key variables (name, age at death, cause of death) were cross-referenced against available sources, and entries with unclear, inconsistent, or insufficiently documented information were excluded. Final automated audit procedures identified missing values, formatting inconsistencies, and potential data quality issues. These steps are consistent with standard data cleaning and validation practices used in observational and secondary data research [10].

Statistical analysis

Descriptive statistics were calculated to summarize dataset characteristics, including counts of cardiovascular and non-cardiovascular deaths, distribution by reported sex, and age at death. Age distributions are reported as mean with standard deviation (SD) and median with interquartile range (IQR). The observed proportion of cardiovascular deaths is reported as a bare descriptor of the constructed dataset. No confidence interval is reported around this proportion: the dataset was purposively curated and not randomly sampled, so an interval grounded in a binomial sampling framework would not have a defensible interpretation. Cohen’s κ was computed for the inter-rater reliability assessment described in the previous subsection. All analyses were performed in Python 3.11 (Python Software Foundation, Wilmington, DE, USA) using the pandas (v2.1) and numpy (v1.26) libraries for data handling, scikit-learn (v1.4) for Cohen’s κ computation via sklearn.metrics.cohen_kappa_score, and matplotlib (v3.8) for figure generation. The full analysis pipeline, including descriptive statistics, kappa computation, and figure generation, is openly available alongside the dataset [8].

The observed proportion of CVD cases is partly a function of investigator-driven inclusion and is not interpreted as an estimate of CVD prevalence among celebrities or a reliable benchmark against population mortality data; comparison with global cardiovascular mortality figures is reserved for the Discussion as a methodological illustration only.

No inferential statistical modeling was performed. Given the curated and non-random nature of the dataset, all analyses were interpreted as exploratory and descriptive.

## Results

Overall dataset characteristics

The final dataset comprised 164 individuals, including 84 cardiovascular deaths (51.2%) and 80 non-cardiovascular deaths (48.8%).

Males accounted for 121 cases (73.8%) and females for 43 (26.2%). Males comprised 77 of 84 CVD cases (91.7%) and 44 of 80 non-CVD cases (55.0%). The distribution by reported sex and cause-of-death category is presented in Table 1.

**Table 1:** Descriptive characteristics of the curated celebrity mortality dataset. Values are counts and percentages or summary statistics. CVD: cardiovascular disease; SD: standard deviation; IQR: interquartile range.

Characteristic	CVD (n = 84)	Non-CVD (n = 80)	Total (n = 164)
Number of individuals	84	80	164
Male, n (%)	77 (91.7%)	44 (55.0%)	121 (73.8%)
Female, n (%)	7 (8.3%)	36 (45.0%)	43 (26.2%)
Mean age, years (mean ± SD)	60.9 ± 18.4	38.6 ± 19.6	50.0 ± 22.0
Median age, years (IQR)	65.0 (46.5-73.2)	32.5 (26.8-44.5)	46.0 (31.0-69.0)
Specific cause description, n (%)	74 (88.1%)	73 (91.2%)	147 (89.6%)
Broad/non-specific description, n (%)	10 (11.9%)	7 (8.8%)	17 (10.4%)

**Figure 1:**
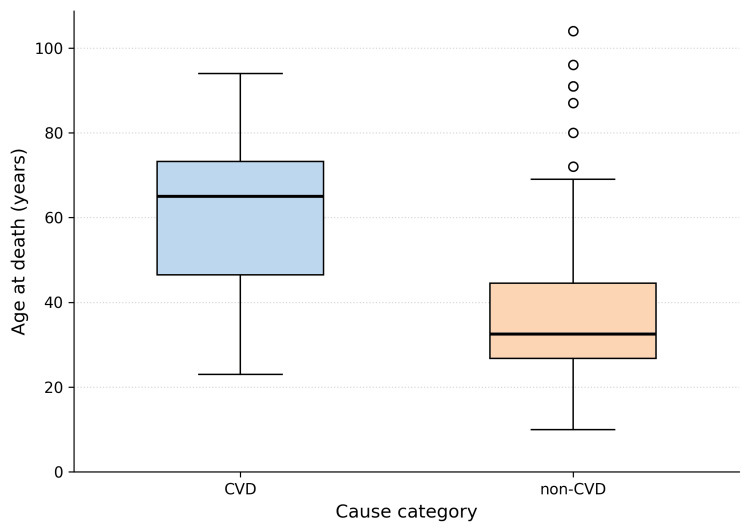
Age at death by cause-of-death category. Box plots display the distribution of age at death for CVD and non-CVD cases. The horizontal line within each box represents the median; boxes indicate the interquartile range; whiskers extend to the most extreme non-outlier values; circles denote individual outliers. CVD: cardiovascular disease.

Mean age at death was 60.9 years (SD = 18.4) for CVD cases and 38.6 years (SD = 19.6) for non-CVD cases; median ages were 65.0 (IQR = 46.5-73.2) and 32.5 (IQR = 26.8-44.5) years, respectively. Distributions are illustrated in Figure 1.

**Figure 2:**
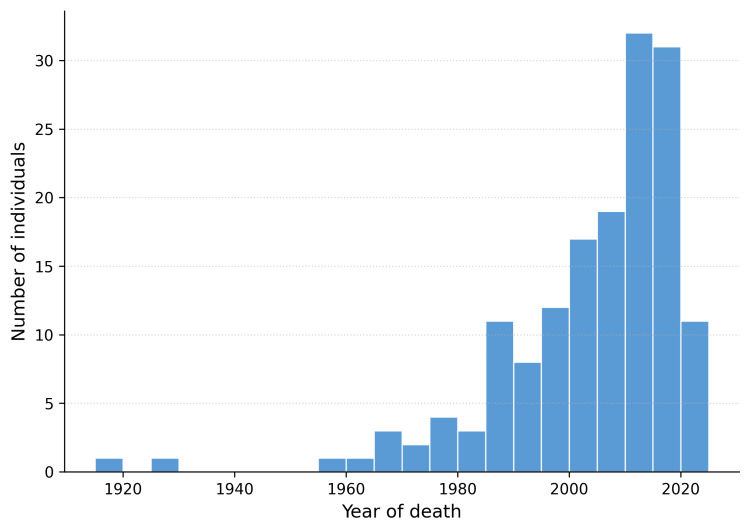
Temporal distribution of deaths included in the curated celebrity mortality dataset. The histogram displays the number of individuals by year of death among the 157 cases with available year-of-death data (recorded years 1916-2023), in five-year bins.

The dataset covered recorded years of death from 1916 to 2023 among the 157 cases with available year-of-death data (Figure 2), with a greater concentration of cases in more recent decades.

Cardiovascular subcategory distribution

Subdivision of the 84 cardiovascular cases into ICD-aligned categories is shown in Table 2. The largest subcategory was heart failure and other unspecified CVD, which comprised 33 cases (39.3% of CVD cases; 20.1% of the full dataset). Ischemic heart disease accounted for 22 cases (26.2%), followed by cerebrovascular disease (nine cases; 10.7%), arrhythmia and sudden cardiac death (seven cases; 8.3%), aortic and pulmonary vascular disease (seven cases; 8.3%), and cardiomyopathy and structural heart disease (six cases; 7.1%).

**Table 2:** Subdivision of cardiovascular deaths (n = 84) into ICD-aligned subcategories, with male predominance and age summaries. CVD: cardiovascular disease; SD: standard deviation; ICD: International Classification of Diseases.

CVD subcategory	n	% of CVD	Male, n (%)	Mean age, years (mean ± SD)
Ischemic heart disease (I20–I25)	22	26.2	21 (95.5%)	60.0 ± 14.7
Cerebrovascular disease (I60–I69)	9	10.7	8 (88.9%)	59.6 ± 16.8
Aortic and pulmonary vascular disease (I26, I71–I72)	7	8.3	6 (85.7%)	49.4 ± 15.0
Cardiomyopathy and structural heart disease (I42, Q20–Q24)	6	7.1	5 (83.3%)	35.5 ± 9.6
Arrhythmia and sudden cardiac death (I46–I49)	7	8.3	6 (85.7%)	67.4 ± 12.3
Heart failure and other unspecified CVD (I40, I50–I51)	33	39.3	31 (93.9%)	67.6 ± 19.4
Total CVD	84	100.0	77 (91.7%)	60.9 ± 18.4

Mean age at death varied substantially across subcategories. Cardiomyopathy and structural heart disease cases occurred at the youngest mean age (35.5 years; SD = 9.6), reflecting cases of inherited or congenital cardiac conditions presenting in early adulthood. Aortic and pulmonary vascular disease cases occurred at intermediate ages (mean = 49.4 years; SD = 15.0), while ischemic heart disease (mean = 60.0 years), cerebrovascular disease (mean = 59.6 years), arrhythmia and sudden cardiac death (mean = 67.4 years), and heart failure and other unspecified CVD (mean = 67.6 years) occurred at older ages.

Cause-of-death reporting specificity

At the field level, 147 of 164 cases (89.6%) carried at least some clinical label for the cause of death, while 17 (10.4%) were recorded with no medically meaningful label (e.g., “natural causes”). By cause category, 74 of 84 CVD cases (88.1%) and 73 of 80 non-CVD cases (91.2%) carried a clinical label (Table 1). Independent of this field-level flag, however, when the broad CVD category was subdivided, 33 of 84 CVD cases (39.3%) were grouped under heart failure and other unspecified CVD because the reported label, while clinically meaningful, did not specify an underlying etiology (e.g., “heart failure,” “heart disease,” or postoperative complications without an identified underlying disease). The field-level flag and the subcategory-level analysis, therefore, capture different layers of reporting precision; the implications of this distinction are addressed in the Discussion.

## Discussion

This study presents an exploratory analysis of CVD representation in a curated dataset of publicly reported celebrity deaths. The patterns observed should be interpreted as descriptive features of the dataset rather than population-level estimates of celebrity mortality.

The 51.2% proportion of cardiovascular deaths observed in the dataset, against a global cardiovascular mortality reference of approximately 32% from the World Health Organization [6], is included here as a didactic illustration of why apparent benchmarking against population data can mislead, rather than as a finding. The numerical contrast is not informative as evidence about CVD in celebrities, for two distinct reasons that operate simultaneously. First, the dataset was purposively curated to include variation in age, reported sex, and cause of death; the 51.2% figure is therefore substantially determined by the inclusion decisions of the constructor rather than an emergent property of media reporting alone. The mechanisms by which non-random sample construction can distort observed distributions are well established in classical epidemiological methodology and have been formally re-articulated in more recent structural frameworks [11,12]. Second, even an unbiased sample of celebrities would not be expected to match global mortality patterns, because celebrities differ systematically from the general population in age structure (older mean age at peak public visibility), socioeconomic status, healthcare access, lifestyle exposures, and geographic distribution. Differences in age structure are particularly relevant, as CVD accounts for a substantial share of mortality among adults aged 70 years and older worldwide [13], such that any sample skewed toward older ages will tend to exhibit a higher CVD proportion than the global average. Disentangling investigator-driven curation from media-driven reporting bias would require a parallel sample drawn under a systematic inclusion rule (for example, all deaths of Wikipedia-listed individuals within a fixed calendar window); the absence of such a comparator is a key limitation of the present study.

Subdivision of the 84 CVD cases into ICD-aligned subcategories yielded a finding that is itself relevant to the dataset-bias argument. The largest subcategory, heart failure and other unspecified CVD, comprised 39.3% of CVD cases and consisted predominantly of clinical syndromes without a specified underlying etiology (e.g., “heart failure,” “heart disease,” or postoperative complications). The prominence of this subcategory suggests that publicly available mortality reporting frequently records terminal clinical states rather than precise underlying causes. This pattern is conceptually analogous to the long-recognized problem of ill-defined or so-called garbage codes in vital-registration data, in which a meaningful proportion of deaths is assigned to terminal events or non-specific conditions that should not be considered underlying causes; such codes substantially affect the comparability and interpretation of mortality statistics if not appropriately redistributed [14,15]. The phenomenon observed in publicly visible reporting represents a distinct, narrative form of reporting imprecision that aggregate cardiovascular statistics tend to obscure. This pattern also highlights an apparent inconsistency with the field-level cause-report specificity variable: although only 10.4% of all entries were flagged as “non-specific” at that level, a substantially larger fraction of CVD cases (39.3%) describes a terminal clinical syndrome rather than an underlying etiology. The two indicators capture different layers of imprecision: the field-level flag identifies cases in which no medically meaningful label was reported at all (e.g., “natural causes”), whereas the subcategory analysis identifies cases in which a clinically meaningful label was reported but did not specify an underlying cause. Future datasets in this area would benefit from operationalizing reporting specificity at both levels separately - distinguishing whether any clinical label was given from whether the label identified an underlying etiology - rather than as a single binary variable.

Demographic structure within the dataset further illustrates how selection processes shape apparent disease distributions. Cardiomyopathy and structural heart disease cases occurred at a notably young mean age (35.5 years) in a small subgroup (n = 6) of a purposively selected dataset; this descriptive observation is consistent with media attention to sudden cardiac death events in young athletes - a phenomenon with disproportionately high public visibility relative to its measured population incidence. Registry-based and prospective surveillance studies of competitive athletes report low absolute numbers of sudden cardiac death events, with hypertrophic cardiomyopathy, congenital coronary anomalies, and related structural conditions among the most frequently identified underlying causes [16,17]. The parallel between the present subgroup and this prior literature is offered as hypothesis-generating rather than as evidence from the dataset itself, given the small cell count and the purposive selection. Conversely, the heart failure and arrhythmia/sudden cardiac death subcategories in the present dataset occurred in older individuals (mean ages of 67.6 and 67.4 years, respectively), consistent with end-of-life clinical syndromes. This non-uniform age distribution across CVD subcategories indicates that summary CVD statistics from curated datasets reflect the visibility of distinct clinical scenarios rather than a representative sampling of cardiovascular mortality.

The marked male predominance of cardiovascular cases in the dataset (91.7% male) parallels but substantially exceeds the male predominance reported for cardiovascular mortality in epidemiological data [7]. Population-level analyses indicate that, although coronary heart disease mortality is consistently higher in men than in women, the magnitude of sex differentials in coronary heart disease and stroke mortality is typically narrower than the disparity observed here and varies considerably with age and across regions [18]. CVD nevertheless remains the leading cause of mortality in women globally, accounting for a major proportion of female deaths, and continues to be understudied, under-recognized, and undertreated in this group [19]. The very high male predominance in the present dataset may plausibly reflect the sex composition of media-visible occupations rather than a difference in underlying disease biology; this interpretation, however, cannot be directly tested with the current dataset, which does not include a comparator that holds occupation or media visibility constant. The observation nevertheless underscores how visibility-driven sampling intersects with disease epidemiology to produce distorted distributions.

Taken together, these findings illustrate a mechanism: distributions observed in curated datasets of media-visible mortality reflect the joint effects of investigator selection, demographic non-comparability between celebrities and the general population, and reporting imprecision in publicly available sources. The dataset provides a concrete worked example of methodological concerns long recognized in the selection-bias literature [11,12], rather than an empirical decomposition of the relative magnitude of any single mechanism. The patterns reported should not be read as evidence regarding the true epidemiology of CVD.

Limitations

Several methodological limitations should be acknowledged in addition to those inherent to the exploratory study design. Case identification, primary classification, and analysis were performed by one investigator (M.A.A.) who was not blinded to the study hypothesis; the dataset may therefore reflect that investigator’s own selection and interpretive biases. To partly mitigate this, inclusion criteria, operational definitions, and the a priori CVD subcategory classification rules were specified in advance, and a second coder (B.A.A.) independently re-classified a random sample of 30 cases drawn from the dataset under blinded conditions; agreement was perfect for both the binary CVD/non-CVD decision and the six-category subcategory decision (Cohen’s κ = 1.00 for both). This reliability assessment supports the reproducibility of the rule-based classification on the subset re-classified, but does not address potential selection bias in case identification, which remains a residual concern; the openly available dataset and classification rules [8] permit any reader to perform further independent re-classification on the full 164-case dataset.

The dataset was purposively curated to include variation in age, reported sex, and cause of death; this selection strategy substantially determines the observed CVD proportion and limits the extent to which the resulting distribution can be attributed to media or reporting bias alone. The absence of a parallel, systematically sampled comparator dataset prevents quantification of the relative magnitude of investigator-driven and media-driven biases, and the present study does not claim to measure either of these contributions in isolation. A prospective case-by-case inclusion log was not maintained during data extraction; the source-list categories consulted, and the structure of the inclusion decisions are described retrospectively in the supplementary materials [8], and prospective inclusion logging is recommended as a methodological standard for future work building on this dataset.

Several further limitations should be noted. The dataset was restricted to English-language publicly available sources, principally Wikipedia and its references; this introduces language, geographic, and Western-media biases that further limit generalizability, as comparative analyses across language editions of Wikipedia have shown that the prominence and ranking of public figures differ substantially by cultural context [9]. Cause-of-death information was derived from secondary reporting and may contain inaccuracies or imprecision. The dataset spans more than a century of recorded years (1916-2023, among cases with available year-of-death data), and diagnostic conventions, ICD revisions, and media reporting standards have evolved substantially across this period; older cases were classified using terms (e.g., “heart failure” and “heart disease”) that have shifted in clinical specificity over time, which both contributes to the prominence of the non-specific CVD subcategory and reduces classification consistency across decades. Older cases are also likely underdocumented relative to recent ones, contributing to the observed temporal skew. Year of death was not recorded for seven cases (4.3%), which are excluded from the temporal distribution but retained in all other analyses. Finally, the modest sample size (n = 164) and the small cell counts in some CVD subcategories (as few as six cases) limit the stability of subgroup comparisons; the subcategory-level age and sex observations should be regarded as hypothesis-generating rather than as established patterns.

## Conclusions

The patterns observed in this curated dataset are partly an artifact of investigator selection, partly a reflection of demographic differences between celebrities and the general population, and partly an expression of reporting practices in publicly available sources. The high proportion of non-specific underlying causes within the cardiovascular category, the descriptive age pattern across CVD subcategories, and the marked male predominance of CVD cases together illustrate the mechanism by which dataset construction and reporting jointly shape apparent disease distributions in media-visible mortality data. The relative contribution of any single mechanism cannot be quantified from the present dataset alone; the manuscript’s contribution is a conceptual and methodological demonstration anchored in an openly auditable worked example, not an empirical decomposition of bias sources.

These findings underscore the limitations of inferring epidemiological reality from publicly visible mortality data. Future research using systematically sampled, population-representative cohorts and parallel comparator datasets would allow the relative contributions of selection, demographic, and reporting biases to be more directly quantified, and would provide a stronger empirical basis for understanding how media-visible mortality data may shape public perception of disease burden.
